# Building towards common psychosocial measures in U.S. cohort studies: principal investigators’ views regarding the role of religiosity and spirituality in human health

**DOI:** 10.1186/s12889-020-08854-8

**Published:** 2020-06-22

**Authors:** Alexandra E. Shields, Tracy A. Balboni

**Affiliations:** 1 Harvard/MGH Center on Genomics, Vulnerable Populations, and Health Disparities, Mongan Institute, Massachusetts General Hospital, 50 Staniford St, Suite 802, Boston, MA 02114 USA; 2 Harvard Medical School, Boston, MA USA; 3 Department of Radiation Oncology and Department of Psychosocial Oncology and Palliative Care, Dana-Farber Cancer Institute and Brigham and Women’s Hospital, Boston, MA USA

**Keywords:** Religion, Spirituality, Chronic diseases, Health disparities, Prospective cohort study, Principle investigator, Qualitative, Interviews

## Abstract

**Background:**

The goal of this study was to understand prospective cohort study Principal Investigators’ (PIs’) attitudes regarding the importance of religion and spirituality (R/S) on disease etiology in order to identify barriers and opportunities for greater inclusion of these domains in high-quality epidemiological research.

**Methods:**

One-hour, semi-structured qualitative interviews were conducted with 20 PIs, who represent 24 different National Institutes of Health (NIH)-funded prospective cohort studies in the U.S. Collectively, these PIs collect detailed health data on approximately 1.25 of every 100 adult Americans. Sample size was calculated to achieve thematic saturation.

**Results:**

The majority of PIs we interviewed viewed R/S as potentially important factors influencing disease etiology, particularly among minority communities that report higher levels of religiosity. Yet nearly all PIs interviewed felt there was not yet a compelling body of evidence elucidating R/S influences on health, and the potential mechanisms through which R/S may be operating to affect health outcomes. PIs identified 5 key areas that would need to be addressed before they would be persuaded to collect more R/S measures in their cohorts: (1) high-quality, prospective studies that include all appropriate covariates for the outcome under study; (2) studies that posit a plausible biological mechanism of effect; (3) well-validated R/S measures, collected in common across multiple cohorts; (4) the need to address bias against R/S research among investigators; and (5) NIH funding for R/S research.

**Conclusions:**

Results of this study provide a roadmap for future R/S research investigating the impact of R/S influences on disease etiology within the context of U.S. prospective cohort studies. Identifying significant R/S influences on health could inform novel interventions to improve population health. Given the higher levels of religiosity/spirituality among minority communities, R/S research may also provide new leverage points for reducing health disparities.

## Background

To date, genome-wide association studies (GWAS) have identified significant loci implicated in a number of highly prevalent and rare conditions, yet there remain relatively few examples of GWAS informing translational medicine [1]. Instead, risk alleles identified in GWAS are now understood to influence disease risk through functional alterations of gene expression levels [2] and complex gene-environment interactions that underlie gene expression [3, 4]. The inclusion of the social environment in gene-environment research, however, has been limited by the lack of common measures of these domains across national prospective cohort studies, even when there is broad agreement on the clinical relevance of the exposure (e.g., stress, quality of sleep, poverty, trauma, early childhood adversity, social support, coping). Complex gene-environment studies also require large numbers of participants for adequate power [5], so it is often necessary to pool data across multiple cohorts. There is thus a critical need to identify the most important social environment measures influencing health and to promote the common collection and use of these measures across prospective cohort studies.

Over the past few decades, a robust body of research has demonstrated the importance of psychosocial stress [6–8] and resilience [9] in relation to disease etiology. One dimension of the psychosocial domain that has been understudied is religion and spirituality (R/S). As of 2014, more than 77% of Americans reported that their religion is very or somewhat important to them, with the figures for African Americans and Hispanics/Latinos even higher at 91 and 84%, respectively [10]. Given that these same communities bear a disproportionate burden of illness, understanding how different dimensions of R/S may support or undermine health may provide new leverage points for addressing persistent health disparities. In the past decade, the number of Americans who report that they are not affiliated with any religion has grown by nearly 30 million, and the number who report never attending religious services has increased [11]. On the other hand, the number of Americans who report being spiritual but not religious increased by 8% between 2012 and 2017. This increase has occurred broadly across the American population, with similar magnitudes of percentage increases occurring among all genders and racial/ethnic groups, and among both democrats and republicans [12]. It is therefore more important than ever that epidemiological reseach investigates a broad and diverse set of factors related to R/S in order to reflect the dynamic and changing landscape of R/S in the U.S., and the influence of these dynamic factors on health outcomes.

The lack of engagement with R/S and health research within the epidemiological research community may be a result of the limited number of robust prospective studies investigating R/S influences on disease etiology [13, 14]. There are, however, a growing number of high quality prospective studies of R/S influences across multiple disease endpoints. To date, studies on church attendance have produced the most robust evidence, with several prospective studies demonstrating significant protective effect of church attendance on mortality and depression [15–19]. Self-assessed spirituality and religious coping have also been found to have significant prospective associations with incident hypertension [20, 21], colon cancer [22], HIV outcomes [23], cognitive functioning and mental health [24–31], change in pulmonary function among adolescents with cystic fibrosis [32, 33], and CD4 levels and viral load among HIV/AIDS patients [23, 34, 35].

The lack of robust and consistent R/S measures across cohorts’ data collection efforts has made it difficult to systematically assess the relative importance of various R/S measures in disease etiology across a range of conditions, compare results across cohorts, and replicate findings. Principal Investigators (PIs) of cohort studies are ultimately responsible for which measures are collected from their study participants. As the final arbiters in deciding which measures get included in each round of data collection, cohort PIs have enormous influence, and shape the practice and culture of epidemiological research. This influence is perhaps greatest with respect to research domains at the margins, such as R/S research, where opinions regarding the value of collecting such measures are more likely to vary. Herein lies the problem for the field. On one hand, additional high-quality, prospective studies are needed to determine the role of various measures of R/S in disease etiology, and yet the range of R/S measures needed to conduct such research are currently unavailable within our nation’s prospective cohort studies. It is within this context that we undertook a qualitative study to assess cohort PIs’ perspectives and attitudes regarding the potential impact of R/S beliefs, practices, and experiences on human health, the mechanisms or pathways through which they imagine R/S may operate to affect disease risk, and the kinds of evidence they would need to see before being persuaded to invest cohort resources in collecting new (or additional) R/S measures.

## Methods

### Defining religion and spirituality

There is a lack of consensus in the field regarding how to define R/S. In this study, we define religion as a shared set of beliefs and practices that reflect a particular relationship to the divine [14, 36]. Examples include beliefs (such as life after death or that God is forgiving), or practices (such as attending church, praying, or meditating). Such items can be measured in a fairly straightforward way [13]. Defining spirituality, on the other hand, is far more challenging. It remains poorly defined in R/S research [37], often intentionally so in order to allow individuals to ascribe their own meanings to the term “spiritual” [38, 39]. Spirituality is generally understood to encompass religious practice and beliefs, but also includes diverse self-definitions of the source of meaning in one’s personal “search for the sacred.” [37] While such an open definition of spirituality makes sense from a theological perspective, it hampers the advancement of empirical R/S research, where terms need to be consistently defined to allow clear interpretation and replication of results. For the purposes of this study, we developed a streamlined definition of spirituality that draws on national palliative care guidelines [39]: “That transcendent dimension of human experience through which individuals seek and express ultimate meaning and purpose in their lives and/or relationship to the divine or sacred.”

### Research team

It is worth discussing briefly the backgrounds and orientations of the authors, so as to make clear any biases they may have brought to the analysis. Dr. Shields also has a Master’s degree in systematic theology and has a vested interest in researching how R/S influence health on a population health and biological level. She directs a research center that conducts transdisciplinary research aimed at elucidating the underlying causes of health disparities, investigating ways to reduce health disparities, and addressing ethical and social implications of genomics research. One of the driving convictions of her center’s research program is that religiosity and spirituality may be important and understudied resources for promoting resiliency and health within minority and other socially disadvantaged communities. Dr. Balboni is a radiation oncologist with a longstanding interest in understanding the ways in which patients’ religious or spiritual beliefs and practices influence their health care decision-making, particularly at the end of life. Spirituality is an important aspect of life for both Drs. Shields and Balboni. Dr. Shields doesn't belong to a particular religious denomination, and Dr. Balboni has experience and familiarity with both the "spiritual not religious" and Christian religious/spiritual traditions.

Despite these personal orientations, we sought to mitigate bias stemming from investigators’ biases by using good interviewing practices in which questions are asked without providing examples or steering the discussion in ways that are apt to introduce bias. Further information on data triangulation is provided in the Data Analysis section, below.

### Participants and recruitment

With respect to developing our study sample of PIs, given our interest in generating new knowledge useful for reducing health disparities, we first developed an initial list of National Institutes of Health (NIH)-funded cohort studies that included large, national samples of racial/ethnic minority communities. Additional cohorts were identified through the published literature, NIH resources, and consultation with epidemiologist advisors to the project. We then developed a ranked list of 30 cohort studies based on the following criteria: [1] racial/ethnic composition of cohort [2]; length of time cohort had received competitive funding (as a proxy for influence of the PI) [3]; clinical conditions covered; and [4] inclusion of a large, nationally representative sample.

This study was carried out as part of a larger project investigating perspectives of PIs on psychosocial factors in general and R/S influences, in particular. PIs were contacted, recruited, and then interviewed about these two topics simultaneously. The results concerning psychosocial factors more broadly are published in a separate manuscript (Argentieri MA, Seddighzadeh B, Philbrick SN, Balboni TA, Shields AE. A Roadmap for Conducting Psychosocial Research in Epidemiological Studies: Perspectives of Prospective Cohort Study Principal Investigators. BMJ Open. 2020; in press.). Contact information for the PI of each study was identified, and PIs were invited via email to participate in this qualitative study. Telephone calls were scheduled with those interested in learning more, during which time PIs were provided with additional information about the study to facilitate informed consent and were again invited to be interviewed then or on a future date of their choosing. PIs who agreed to be interviewed were offered a $100 honorarium as a token of appreciation for their participation. We followed these procedures until we reached our study goal of 20 PI interviews. Neither of the study investigators had a previous relationship with any of the PIs contacted. Only one PI with whom we discussed the study declined to participate. All but two participating PIs refused the honorarium. Institutional Review Board (IRB) approval for this study was obtained from the Partners Human Research Committee (PHRC). Verbal consent was given by participants, although this study was deemed to be exempt from continuing IRB review by the PHRC.

### Data collection

One-hour, semi-structured interviews were conducted with each participating cohort PI by the Principal Investigator of our qualitative study (AES) or jointly by two members of the study team (AES and TAB, both female PhD-level research investigators) in 2014–15. A semi-structured interview guide was developed by the study team, with input from several investigators participating in the National Consortium on Psychosocial Stress, Spirituality, and Health (CoSSH). Questions addressed in the interview guide included: (1) PIs’ experiences with and exposure to research on R/S; (2) their assessment of the importance of R/S practices and beliefs in understanding disease etiology; (3) reasons why they decided to collect the R/S measures their cohort has collected thus far, or for not collecting any R/S measures; (4) their assessment of the quality of existing R/S research; (5) their beliefs regarding the pathways or mechanisms through which R/S might operate to affect human health, if at all; and (6) the kinds of evidence they would need to see before being willing to invest cohort resources in collecting new or additional R/S measures going forward. Depending on the flow of each interview, the order in which these areas where discussed varied. Based on our team’s previous work [40–45], we anticipated that 20 individual interviews would be more than sufficient to achieve thematic saturation.

### Data analysis

All interviews were recorded and transcribed. Drs. Shields and Balboni later reviewed the transcripts for accuracy. Interviewers’ notes from the interview not only captured initial impressions, but also provided another source of data useful for addressing instances where the audio recording was difficult to decipher. Interview transcripts were analyzed using a grounded theory approach [46, 47]. The faculty interviewers and two Master’s-level research assistants (RAs) coded 40% of transcripts and identified key themes. Coding discrepancies were addressed through discussion, comparison of the raw data, and refinement of code definitions. The interviewers then finalized the preliminary coding scheme. The remaining transcripts were coded independently by the RAs, using Atlas-ti software (Version 5.0). Data were analyzed using content analysis to identify major concepts, and axial coding to group and connect related data [45, 47, 48]. Within each topic area, we highlighted statements characteristic of the majority of those interviewed, as well as statements from those with divergent views. The quotes included in this report are illustrative of sentiments expressed by several PIs, unless otherwise noted. No repeat interviews were carried out, and participants were not provided with transcripts or findings to provide comments or feedback.

Many steps were taken to maximize dependability (consistency, reliability) and credibility (the truth of findings, internal validity) of study conclusions [49]. We incorporated triangulation at two levels: [1] involving a multidisciplinary research team in coding and analysis (investigator triangulation); and [2] including PI participants from diverse communities and disciplines whose cohort studies include participants from diverse racial/ethnic communities and geographical regions of the country (data triangulation). The Kappa score for assessing congruence of coding between coders was 0.95.

## Results

**Fig. 1:**
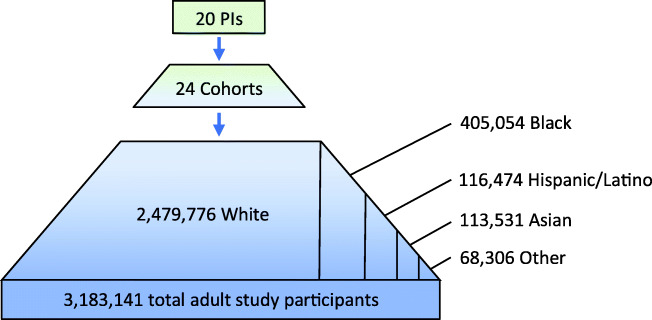
Total number of study participants represented by participating PIs’ cohorts, by race/ethnicity

The final study sample of 20 PIs represented 24 different cohorts, and included men and women from several different racial/ethnic communities (approximately one-quarter were from minority communities). They represented a wide range of ages, although few were younger than 55 years old. Epidemiology was the academic discipline of the vast majority of those interviewed, although a few were trained in medicine or other disciplines, or had training in more than one discipline. The public health research infrastructure overseen by this relatively small group of PIs is enormous. Collectively, the 20 PIs interviewed for this study represent longitudinal health data on nearly 3.2 M individuals, or roughly 1.25 out of every 100 adults in the U.S. aged 18 or over. This includes data on approximately 400,000 African Americans and 120,000 Latinos, as well as many other ethnic communities (Fig. 1). Below we report on findings with respect to key questions asked of PIs. Supplementary Tables 1–4 summarize key themes identified and provide a representative quote for each theme.

### Importance of religion and spirituality to understanding human health

We began our interviews by asking PIs how important they believe religion and spirituality are to understanding human health and risk of disease. We provided the working definitions of R/S noted above. Of the 20 PIs interviewed, nine felt that R/S are important psychosocial influences that likely have a significant positive impact on health. Another eight PIs were open to the possibility that R/S influences could be important, but believed “the jury is still out.” Only three PIs believed that R/S are not important constructs to study in understanding human health.

#### Positive views about the importance of R/S in understanding human health

Five sub-themes emerged regarding PIs’ positive views on R/S and health. These included: (1) R/S as a critical locus for resilience and personal identity; (2) R/S as an important source of communal support; (3) R/S promoting healthy behaviors; (4) R/S being especially important in minority communities; and (5) barriers to R/S research.

PIs most often connected R/S as important resources for resilience in coping with stress, but also alluded to a powerful, transcendent dimension of life and identity that is difficult to measure. This reflection on spirituality by one epidemiologist is illustrative of the PIs who held this view:

As an epidemiologist, I think of it [spirituality] primarily as a modulator of stress, but it’s more than that. There are positive social networks, and support, and healing connected with [spirituality], and sort of self-affirmation about who I am, what I’m doing, that almost for sure have physiological effects. So I look at [spirituality] as part of complex physiological networks dealing with pathways that are almost for sure related to cardiovascular disease.

PIs also focused on R/S as integrally linked to community and social support. As one PI said, “My personal sense is that [spirituality] is an integral part of life, which might also link into social support, because it’s hard to separate the two.” This PI described spirituality and social support as “two key sorts of primordial factors associated with disease outcomes.” Others viewed religious communities as providing critical social support based on their own experience within religious communities, regardless of their own religious beliefs. As one self-described agnostic Jew described in explaining why she believes R/S “definitely affect health”:

The social support people get from church is tremendous. My own personal experience [as someone who is agnostic] makes it hard for me to judge what the added coping and support and well-being people get from their religious beliefs is, as opposed to the social framework. But for me, you know personally, going to a synagogue when I was [experiencing a personally difficult time] was a tremendous social boon … And so I do believe that the support that comes from that allows people to cope better with challenges, to be less overwrought, to help them get things done, to reinforce messages about healthy lifestyle. So you know, from that perspective, I think it’s a no-brainer [that R/S affects health].

A third group of PIs viewed R/S as influencing health primarily through promoting healthy behaviors, such as diet and exercise, and curbing unhealthy behaviors, such as smoking and alcohol use:

Seventh Day Adventists have a whole pattern of behavior based on their religion that influences their risk of disease, as do people of Eastern sects, in Hindus and other groups, have very specific sorts of prescribed behavior patterns based on religion. So it can be very powerful.

A fourth important sub-theme was PIs’ belief that the impact of R/S on health likely differs across racial/ethnic communities. Several PIs believed that R/S influences might be especially important to study in African-American or Hispanic/Latino communities, where religion plays an especially prominent cultural role. Regardless of their own personal R/S beliefs or practices, PIs whose cohorts had large numbers of minorities were especially likely to believe that R/S are important influences on health. One PI whose cohort included large numbers of African Americans explained:

I think it [religion and spirituality] would be more important to study in minority groups, who are under other stressors … We’re putting more emphasis on that [religion and spirituality] as factors that moderate stressors or factors that could turn things around, and thus improving the quality of life or life expectancy, or even prevent the recurrence of certain diseases … So for African Americans, religious activities are really, really important.

Lastly, nearly all of the nine PIs who reported believing that R/S likely had important influences on health noted dissatisfaction with the quality of existing R/S research, while also noting barriers to pursuing high-quality epidemiological research on R/S and health. Some had even engaged in R/S research, but had experienced a bias against R/S research among their colleagues. One PI described sharing some interesting preliminary results from a pilot study on R/S, but found her colleagues decidedly unenthusiastic about pursuing this area of research: “So though the question of spirituality might seem reasonable to us, spirituality is something that many members of my team might go, ‘Blah!’”

Nearly all PIs who believed R/S are important influences on health discussed the limitations of existing R/S measures, seeing them as “all over the place,” lacking rigorous validation, or being insufficient to capture such a complex phenomenon. Despite their criticisms, PIs were very engaged in thinking about what they saw lacking in existing measures, often offering recommendations for how to improve them. For example, one PI suggested a shift in focus of R/S measures:

I think the types of questions where you get at *how* R/S affects people, like strength and comfort questions – where you get at the *resulting feelings* from your religious participation – I think those are probably more important than your *habit* of going or not going to church, which can just mean a lot of different things.

PIs also noted the lack of consistent measures across cohorts, making replication of results in other cohorts impossible.

#### Equivocal views regarding the role of R/S in health

Eight of 20 PIs interviewed believed that R/S influences may significantly affect health, but felt “the jury is still out.” Sub-themes within this larger theme included: [1] insufficient evidence to determine if R/S influence health, and [2] the lack of a sufficiently nuanced conceptual framework that addresses the complex ways in which R/S may affect important health outcomes. They emphasized the lack of high-quality (i.e., prospective) studies demonstrating that R/S does indeed have a strong direct or modifying effect on disease etiology. Of particular conern were the lack of longitudinal studies that included the full range of expected covariates for a particular clinical condition, and the lack of replicated results. PIs in this group were not dismissive of R/S research, but simply felt that the field of R/S research had not yet produced the quality of evidence that the epidemiological community would find compelling:

I think the findings on spirituality measures have been sort of mixed in relation to disease outcomes. Well, that’s the nature of our field, isn’t it? [laughs] A lot of factors, when you look at the data, are mixed. And it’s only after a crucial body of evidence has emerged that you’re able to look at the totality of studies... So right now, the field is not mature. You don’t have a lot of studies in this area, and findings are mixed.

Many PIs also believed that the field had not yet presented a robust and cohesive conceptual framework for research on R/S and health, pointing to a failure to study R/S influences in conjunction with the biological, environmental, and other psychosocial factors already established as affecting the etiology of a particular disease. They emphasized the need for R/S research to both include biological variables in analytic models and conceptualize R/S influences within the context of known biological pathways or mechanisms at play in the studied disease. One PI explained this challenge, using the example of cardiovascular disease:

As you know, CVD is a lifecourse disease, and it’s a lifestyle disease … we understand all this is a constellation of biological phenomena that translate into an event on a particular day, in a particular time, in a particular moment in a person’s life. But, you know, it follows if you look back – there’s a cascade of events, which begins with the genetics that you are born with, and a series of environmental factors that you’re exposed to, including the built environment, and including your socioeconomic position over the lifecourse. And on top of that, a number of bio-behavioral risk factors. So religiosity is one of a gamut of [influences] on a complex risk factor. So I think one of the challenges is going to be to build a framework, a thesis, or a scientific model that places religiosity in the context of a lot of other pathways that eventually condenses into a biological model.

#### Beliefs that R/S are not importance influences on human health

Only a handful of PIs (3/20) believed that R/S influences were simply not important domains to study in health research. Subthemes included: (1) a lack of compelling research demonstrating importance of R/S in understanding health; (2) beliefs that R/S measures are merely proxies for other, more critical factors influencing health; (3) an inability to identify plausible mechanisms through which R/S might influence health; and (4) an inability to imagine how R/S research could generate new knowledge that could lend itself to interventions.

While this small group of PIs similarly criticized the quality of R/S research to date, these PIs tended to express a general discomfort with the domain of R/S in general, often because they were not spiritual persons themselves. As one PI explained, “As somebody who has a hard time picturing what it means to be praying, it’s hard to know what those many hours are doing...I just don’t know what it’s a measure of.”

To the extent that R/S influences were shown to be significant, such as church attendance, they believed that any potential influence of R/S could be better captured by other measures that focused on the downstream effects of individuals’ spirituality, such as social support or stress reduction. As one PI explained:

I don’t feel like, personally, that these [R/S] variables are very important variables to be looking at – if anything, they are proxies of something else that I would prefer to get at. So if they’re proxies of social support, I’d prefer to get a measure of social support. If they’re proxies for stress mitigation, I’d like to get at more of a proxy of stress.

This group of three PIs had difficulty envisioning a potential *biological pathway or mechanism* through which spirituality might affect disease etiology, and thus they viewed R/S as biologically irrelevant. A PI whose work focuses on cancer captures this sentiment: “This exposure couldn’t possibly cause or protect one from cancer, therefore we don’t see this [the role of R/S in health] as a reasonable scientific question.”

Like other groups, these PIs had difficulty imagining how R/S research could be translated into public health interventions, but were consequently pessimistic about R/S research. One PI, for example, despite acknowledging the robust studies on church attendance and mortality, stated, “I mean, what are you going to do? Prescribe that people start going to church?!” The inability to envision how knowledge about R/S and health could be translated into appropriate interventions greatly reduced the value of R/S research among this small group of PIs.

### Rationale for collecting extant measures of religion or spirituality

Although there are relatively few R/S measures collected by U.S. prospective studies to date, 17/24 cohorts represented by this group of PIs had collected measures of religious denomination or religious service attendance in the past, but few had collected any additional measures. We explored with PIs their rationale for collecting the R/S measures they had collected to date, or reasons why their cohort has not collected any R/S measures.

#### Reasons why PIs collected the R/S measures they did

Among those whose cohorts had collected previous R/S measures, three subthemes emerged: (1) study teams were persuaded by recent literature; (2) measures collected reflected interest of a particular team member; and (3) R/S were known to be important to the particular ethnic communities represented in their study.

PIs who were persuaded by the literature universally cited studies demonstrating a significant inverse association between church attendance and mortality [15, 17]. Some also emphasized hypotheses that R/S influence healthy lifestyle behaviors, which leads to better health outcomes:

You know, the other concept that we had, which I think is still valid, is that religion could be considered in one sense to be the parent, or if you like, to be *behind* behaviors. In other words, religion is driving the dietary behaviors in the path, which in turn results in some differences in chronic disease.

Second, PIs reported that the decision to collect particular R/S measures was due to the advocacy of a particular team member, or even a doctoral student, who wanted to study R/S influences on health and had succeeded in making the case to add R/S measures to their survey. One PI explained:

In every case, you have advocates. You have folks who say, here’s our rationale. Here’s why we think it’s important … And, you know, just thinking about quality of life, thinking about social support, thinking about the importance of spiritualty in a global sense, of how it would contribute to us really understanding not only the sort of overarching factors associated with things like cardiovascular disease, but also recognizing that we have a very diverse cohort in which spirituality may mediate or may be protective differentially in one of our racial/ethnic groups. So that’s the overall rationale for inclusion of spirituality [measures].

Lastly, PIs explained that they had collected measures of R/S previously because of the importance of R/S to particular communities included in their cohorts, especially in cases where cohorts included a sizable proportion of African Americans and/or Hispanics/Latinos. One PI whose cohort included large numbers of African Americans explained that including measures of spirituality “was pretty important to do for any study that was thinking about getting a thorough assessment of African Americans’ health and disease.” She continued:

I think again, you can’t speak for all African Americans, but in general, particularly in the South, you are speaking about a society that sees religion and spirituality as a central aspect of life. And that, I think, has been true for generations, and is impressively true today. I think if you were to ask 100 African Americans in the South whether they attend church regularly, I would say that 85 of them would say yes … And I think most feel that … one of the great methods of dealing with stress is to turn to turn to God, to turn to a higher power … These are very, very important ideas and ways of life, really. And [African Americans] do see them [R/S] as ways of coping, but they see, beyond that, that this is fundamental to how life should be lived.

Other PIs whose cohort include racially diverse participants echoed this sentiment. One explained:

We have … African-Americans on our advisory board who have expertise in various areas. And we also have some Black investigators, and some Black staff, and even if we hadn’t, we would have been well aware of the fact that in this country anyway, African-Americans tend to be religious, or to take part in religion. That’s a big part of their lives. And we’re aware that there’s a body of literature suggesting that religiosity or spirituality may be protective against the occurrence of certain diseases, or if not against the occurrence, it might be good for prolonging survival. So for all those reasons, we were interested in having some questions on spirituality or religious practices in [our cohort study].

#### Reasons for not collecting R/S measures to date

In exploring why certain cohorts had *not* collected any R/S measures thus far, 4 sub-themes emerged: (1) R/S measures did not “meet the bar” for inclusion in epidemiological research; (2) study team’s lack of knowledge about, or self-proclaimed bias against, R/S research; (3) difficulty imagining how research demonstrating an effect of R/S could be translated to interventions; and (4) lack of NIH funding in this arena.

PIs whose cohorts had not collected any R/S measures to date viewed R/S research as adding little explanatory power beyond that provided by social support measures, as captured in this comment: “You’d expect social support to have an impact on a number of domains. But, from my vantage point, I’ve not seen evidence that [R/S] is at least as strong at this point in time.” The robustness of R/S measures in the field were not seen as “meeting the bar” for inclusion in cohorts’ national surveys. As one PI explained:

I work in the large population arena. That doesn’t mean that everybody has to. And there’s certainly insights that can come from small populations, but to be able to be translated into large population research, the [R/S] field has to evolve to the point where we can actually capture what the experts in the field feel are the true measures of those domains in a rigorous way, and yet, in a way that’s really convenient.

Again, several PIs talked about their own bias or the bias of others against R/S research. They described being uninterested in R/S research because they could not imagine how results showing a protective effect of R/S on health outcomes could be applied to improving population health, and thus concluded that the domains of religion and spirituality were “outside the scope of public health.” As one PI described:

With [R/S research on church attendance], I just wonder what the message is...is the message that people should find God? Or go to church more often? From a personal background, I would feel uncomfortable with public health messages that had to do with religious matters.

Lastly, PIs whose cohorts had not yet collected any R/S measures addressed the lack of interest in R/S among funders, particularly NIH. They described how Requests for Applications (RFAs) influence the kinds of research conducted, particularly in times when funding is tight, explaining that if R/S research was not a priority for NIH, researchers would not study it. As one PI put it, “We had no funding for them (R/S measures); nobody was writing a specific grant related to those hypotheses.”

### Likely pathways or mechanisms through which religion and/or spirituality operate to affect health

Among 17 PIs who believed R/S are potentially important constructs in understanding disease etiology, we explored the various biological mechanisms or pathways through which they believed R/S might influence health. By far, the most common mechanism discussed was R/S as a vehicle for garnering social support and coping with stressful life situations. As one PI said, “I think we are talking about things that are generally incorporated into the stress pathway, and so I would think of the autonomic nervous system. I would think of the HPA axis right off the bat.” Another PI elaborated on this perspective, rooted in his cohort study’s findings:

Probably the most immediate and obvious is related to the stress pathways. And they are related to physiology all over the body – immune function, endocrine function – that would potentially interface with pathways related to cancer, but not as directly as to cardio-metabolic outcomes, one would think. So cortisol, epinephrine, those pathways would be presumably the most immediately directly affected [by] it [R/S], whether through hypothesized immune markers, which do seem to be related, secondarily at least, but perhaps primarily to the stress pathway.

Another PI put it this way: “I think it [spirituality] might be a modifying factor or a mediator. Like social support, for example, mediates certain biological relationships, or even genetic relationships.”

Some PIs were convinced that R/S has an effect on coping with stress through their own experience with chronic disease patients. One PI who had participated in several different studies with diabetes patients offered this observation:

My personal view is that [religion and spirituality] do reduce the stress tied to [illness] when you are, when you surrender to the notion that there is a plan. No matter what happens, there is a plan that you may not fully comprehend, but you put your trust in a higher power that things will be OK.

The second most-cited mechanism or pathway PIs envisioned was that R/S may affect health-related behaviors, such as motivating individuals to refrain from smoking or drinking, or encouraging healthy behaviors, such as exercise or healthy diets. They viewed this behavioral mechanism in two ways, which we have found helpful to conceptualize as: a “proscriptive function of R/S” – delineating acceptable behavior – or an “affirmative function of R/S,” helping people believe that they are *worth* taking care of as shown in religious teachings (e.g., they are “children of God,” or their body is the “temple of God”). One PI describes the proscriptive function this way:

The jury is still out [regarding how R/S affects health], but I think that one can pretty reasonably postulate that some of these [R/S beliefs or traditions] might be driving dietary behavior and so may not be confounders in that sense, but rather the dietary behavior might mediate, in part, the effect of religious variables.

Another PI articulated what we call the “affirmatory function of R/S,” encouraging a sense of self-worth and appreciation for life:

I think it’s well accepted that behavior is critical, and behavior is rooted in something. And if people are spiritual and feel that, one, that their personal existence is important to them, and two, [their personal existence] is important to others, that they’ll often feel more of a responsibility to take care of what they’ve been given...that they see life as a gift, then they are more likely to respect that gift.

Several PIs felt that R/S are important psychosocial constructs, but had more questions than answers regarding potential mechanisms or pathways through which R/S might affect health:

What actually is it that’s helping them? Is it the fact that they have a spiritual leader who can help them with their problems? Is it that they have people that they’re seeing, and it’s simply this group kind of support that’s helping them? … Or is it actual belief in God actually changes something in the brain, or wherever? … Well, [stress] is certainly one pathway. But I don’t know enough about the biology to express an opinion about what other pathways may be involved. But there probably are other pathways. I mean, there always do seem to be multiple pathways.

### What would PIs need to see to be persuaded to collect new or additional R/S measures?

We concluded the interviews with a discussion of the kinds of evidence that PIs would find persuasive in demonstrating that R/S significantly affect health, and thus would encourage them to collect new R/S measures in the future. While PIs’ attitudes towards R/S research varied, all PIs interviewed articulated a strikingly similar set of criteria that R/S researchers would need to meet before prospective cohort PIs would be persuaded to incorporate R/S measures into ongoing data collection efforts, identifying five key themes: (1) the need for high-quality, prospective studies that include all appropriate covariates for the condition under study, replicated in at least one other cohort; (2) the need to posit a plausible biological mechanism through which R/S operate to affect health outcomes; (3) the need for well-validated R/S measures, collected in common across multiple cohorts; (4) the need to address bias against R/S research within the epidemiology research community; and (5) the need for NIH funding for R/S research.

#### High-quality, prospective studies replicated in at least one other cohort

The most foundational requirement cited was that more high-quality, prospective studies of R/S be conducted that demonstrate a significant influence of R/S on important health outcomes. PIs also emphasized that these analyses need to include the full range of covariates expected in high-quality studies of a given condition. Few PIs were aware of extant prospective R/S studies meeting their bar for high quality beyond those associating church attendance with reduced mortality, and characterized existing R/S research as being dominated by cross-sectional studies with variable findings. As one PI put it, “Any more association studies will just add to the confusion.”

#### Posit a plausible biological pathway or mechanisms through which R/S affect health

Virtually all PIs emphasized the need for R/S studies with significant results to at least posit, and eventually demonstrate, a plausible pathway or biological mechanism through which R/S operate to affect the etiology of disease. As one PI plainly stated: “We need studies that have some kind of biologically relevant hypothesis.” One PI who studies cancer captures this emphasis:

What would be very persuasive to me would be some well-designed research that showed biological changes, or some physiologic effects, that were associated with people’s religion or spirituality, on pathways that were meaningful for cancer – cancer pathways.

PIs noted the increasing importance of biomarkers in understanding disease etiology, as biomarkers were often seen as the critical link between the social constructs measured and the biological mechanism hypothesized to be at play. Incorporating biomarkers into R/S research would also demand a significant investment of resources. One PI explained that “what is needed are multiple questionnaires over time [that include R/S measures], with multiple blood samples that parallel it [to support biomarkers].”

#### High-quality, well-validated measures collected in unison across cohorts

One of the greatest challenges in advancing research on the role of R/S in health, voiced by the majority of PIs, was the difficulty of adequately defining the complex dimensions of R/S. They expressed a need for well-validated measures of R/S that are easy to collect across cohorts in order to facilitate replication of results. Here one PI discusses the challenge of measuring R/S influences compared to other more easily quantified influences on health, and the relationship between how easy a measure is to collect and how much it is studied:

For example, coffee is a habitual activity that a large number of people partake in. Very, very easy to measure, and thus we’ve got dozens or hundreds of studies on coffee! – almost every epidemiologist looking for something that’s either protective or is caused by coffee, though there’s no findings there... It’s easy to ask somebody what their coffee habit is. But it’s very hard to get at these kinds of things [R/S influences] … This [R/S research] is so ripe for epidemiological discovery if you can figure out how to measure it.

All PIs interviewed had been engaged in consortia that conduct pooled analyses across multiple cohorts, and experienced the problems of having different measures of the same construct across individual cohorts. Often, constructs that are not available across cohorts get dropped from the analyses altogether. If there are some measures across cohorts, but with varying levels of nuance, the measure is often reduced to the lowest common denominator of measurement. As this PI warns:

You’re going to lose quality if people don’t ask the question in a manner that you can pool across studies. We’re seeing that in everything we do right now, you know? And sometimes we get down to just yes/no when we think about exposure to carcinogens, or hormones, or even diet, whatever. We get down to yes/no and we don’t get anything about dose, dose intensity, because everybody asks it differently.

Several PIs also pointed out that the need for a sufficiently robust body of high-quality research before it makes sense to move towards consensus regarding which measures are important to collect. Timing is important. As one PI explained:

Well, you know, there are reasons to move towards consensus, because it makes it a lot easier to harmonize the data. And so, that’s the pro. The con is, if you don’t choose the right set of questions, then there really isn’t a fit. So I think it’s a bit early to be moving towards a consensus [on R/S measures]. I think that first we need to have some more actual studies that provide results.

#### Address bias against R/S research among epidemiologist

Several PIs also noted that even if a body of high-quality R/S research evidence from prospective studies “met the bar” of world-class epidemiological research, there would still be bias against R/S research within the epidemiological community. PIs offered various explanations for such bias: “For one thing, because most of the investigators are biologically inclined – you know, to look for more biological or genetic explanations of things.” This PI further believed that this focus on biological causes of human disease was likely due to the lack of religiosity among PIs themselves: “My anecdotal impression … is that they [PIs] are not religious. Probably there’s a bigger percentage of naysayers [about the importance of R/S to health] among scientist investigators than maybe other groups of people.” Others noted a tendency among many epidemiologists to view R/S research, along with other psychosocial research, as “soft science” unlikely to have a significant clinical impact. Some PIs believed the lack of R/S research was “just not on our colleagues’ radar,” even when studying populations known to have high levels of religiosity:

I mean, there is a group of people – the behaviorists – who are particularly interested in [R/S]. But it’s not very high on the radar, which I have to say is actually surprising, considering that, in the Hispanic community, for example, two-thirds [of the community] consider their religion either very or extremely important. It indicates that in terms of the population we’re studying, it is important. But the -- the researchers don’t give it the commensurate weight in looking at it research-wise.

#### Increase the availability of NIH funding for R/S research

Finally, PIs emphasized the central importance of NIH funding to support R/S research. Without sufficient interest and commitment of NIH Institutes to advance research in this area, it is unlikely that PIs will use the limited space on their survey instruments to collect R/S measures. Prospective cohort studies are extremely expensive and mostly NIH-funded, and thus very driven by the research agenda advanced by NIH through various topical RFAs. Nearly all PIs interviewed were operating their research groups entirely on grant funding. With a difficult funding environment for researchers whose salaries are completely grant-funded, it is a practical concern to take their cue from NIH and invest in areas of research where future NIH funding is likely going to be awarded. As one PI explained:

“I’m not sure if people would fund it (research on R/S and health). And when I say, ‘fund it,’ I mean at NIH. I’ve never applied for funding anywhere except through NIH. So I think it’s hard to come by … I think many of us would be interested in doing it [R/S research] if we could, but the resources are hard to come by.”

## Discussion

Within the broad domain of psychosocial influences on health, research addressing the impact of R/S on health has received relatively little attention in epidemiological research, despite recent calls for R/S to be recognized as important domains in public health research [50]. The public health “cost” of not appropriately assessing psychosocial influences on health, and more specifically R/S influences, is likely to differentially affect those in low-income and minority communities – groups with higher levels of religiosity. The relatively small number of prospective R/S studies to date is largely due to the lack of R/S measures collected within our nation's prospective cohort studies. Cohort study PIs have enormous influence in shaping epidemiological research, yet little is known about their perspectives on R/S research.

In this first national study of PIs’ attitudes and beliefs regarding the role of R/S in health, our interviews with 20 PIs of NIH-funded cohort studies revealed that approximately half believed R/S are likely important influences on health, viewing R/S as a critical locus for resilience, personal identity, and communal support that promotes healthy behaviors. These PIs also believed that R/S are especially important to study in minority communities. More than one-quarter believed R/S influences might be important, but believed “the jury is still out,” and only three PIs believed R/S are not important influences on health. Even PIs who did believe that R/S influences are likely important to understanding human health thought that the evidence base provided by R/S research to date was lacking, and articulated the need for more robust studies, with results replicated in additional cohorts, and a coherent biological model of influence articulated. There were a range of explanations for why particular cohorts collected the R/S measures they have collected to date, including references to prior studies (e.g., associations of church attendance with mortality), advocacy by a member of their team for a particular R/S measure to be collected, or importance of R/S to minority communities in their cohorts. Reasons most often cited for not investing in the collection of [more] R/S measures included the lack of a body of scientific evidence to date demonstrating significant R/S influences on high priority conditions and the lack of NIH funding for R/S research. Interestingly, with few exceptions, PIs were familiar with relatively few R/S measures or extant prospective studies of R/S and health, with the exception of  studies addressing the role of religious service attendance or being part of a religious community, as opposed to a wider array of spiritual beliefs, practices, and experiences.

Despite diverse attitudes and beliefs regarding the importance of R/S as domains to study in health research, PIs were in almost complete unison in articulating the kinds of evidence they would find persuasive in judging R/S research to be important and thus influence their willingness to collect more R/S measures in their cohorts. Specifically, PIs called for more high-quality, prospective studies that include the full range of covariates that experts in a clinical area would expect to see, that are replicated in additional cohorts, and that posit a plausible biological mechanism or pathway through which R/S influences found to significantly affect health outcomes might be operating.

The limited number of R/S measures currently available within our nation’s prospective cohort studies makes it difficult to meet PIs’ foundational bar of conducting prospective R/S studies and replicating results in additional cohorts. In a recent analysis we undertook investigating the availability of R/S measures in 20 U.S. prospective cohorts, for example, while 13/20 and 10/20 cohorts collected church attendance and religious denomination, respectively, fewer than 5 of the 20 cohorts collected more diverse and functional measures of R/S, such as participation in church social groups (4/20), finding strength or comfort in R/S beliefs (4/20), or using R/S to cope with stressful life situations (2/20). Beyond these, no other R/S measures have been collected by more than 2 out of the 20 cohorts. Detailed information on which R/S measures have been collected across U.S. cohort studies is available through our online R|S Atlas tool (https://atlas.mgh.harvard.edu). While other national surveys collect R/S measures from large national samples over several years, these datasets often do not include the full complement of clinical covariates previously established as affecting risk of a particular disease outcome (these covariates will differ by condition), which are essential to conducting a high-quality epidemiological assessment of R/S influences on risk of developing that outcome.

There are further challenges in demonstrating a plausible biological pathway or mechanism through which R/S operate to affect health outcomes. Not only do R/S researchers need the R/S measures they wish to study to be available in prospective cohort studies, but analyses investigating biological pathways typically require biomarkers that reflect activity within the hypothesized biological pathway of interest, and these are expensive to collect (e.g., ~350/sample for the Illumina EPIC array or ~$700/sample for the SOMAscan proteomic assay). Without using cohort study data where biomarkers have already been collected, or receiving NIH or other grant funding to create the biomarkers needed, it will be extremely difficult for researchers to conduct these more sophisticated analyses on the role of R/S in human health, which have become *de rigueur* in epidemiological research.

One striking finding from our study is the extent to which the selection of measures to be collected by cohorts is a nonlinear process, determined by the interests and biases of particular research teams. The field of research on the role of R/S in disease etiology is in a fairly nascent stage, and thus needs champions within established epidemiological cohorts to convince colleagues to commit resources to collecting the R/S measures and biomarkers needed to test R/S hypotheses and build the evidence base PIs have called for.

Our study has several limitations that should be mentioned. First, we focused on PIs in this study. The field of epidemiological research on R/S cannot advance without the availability of a broader array of R/S measures available within our nation’s prospective cohort studies, and PIs are the key decision-makers who determine which measures their cohorts collect. Our study is thus very “top down” and does not include the views of other study team members who also influence which measures will be collected on each round of data collection, nor end users of cohort studies’ data or members of communities represented by different cohorts. Incorporating the perspectives of this broader array of actors was beyond the scope of the present study. Second, as with all qualitative studies, this is not a systematic empirical assessment of PIs’ attitudes towards R/S research that can be generalized to the community of PIs of cohort studies nationally. Rather, our goal was to deeply explore the attitudes, beliefs, and perspectives of a sample of 20 NIH-funded cohort study PIs – all leaders in the field of epidemiology – regarding their views on the influence of R/S on health and what gives rise to these perspectives. While the 20 PIs interviewed represent diverse ethnicities, ages, and clinical domains of interest, they may not fully capture the views of cohort PIs nationally. According to NIH institute websites, 61 cohorts studies are currently funded by NCI and 9 are funded by NHLBI. The PIs participating in this qualitative study represent roughly a quarter of all NIH-funded cohorts. Future research could survey all NIH-funded cohort PI and investigative teams to quantitatively assess a broader array of perspectives. Lastly, while many PIs offered information about how their own religious/spiritual experiences shaped their views, we did not directly query PIs about their own religious beliefs and practices or spirituality more generally, anticipating that PIs could find such questions offputting. Rather, we sought to engage them in a discussion of R/S measures as one of many kinds of psychosocial measures that may affect disease etiology. Our data therefore cannot support analyses addressing the ways in which PIs’ personal R/S beliefs or practices shape their views regarding the likely importance of R/S in understanding human health, although some individual PIs did offer examples of how their own spiritual experiences have shaped their views.

Despite these limitations, this study provides the first assessment of prospective cohort study PIs’ attitudes and beliefs regarding the influence of R/S on disease etiology, and identifies challenges for the field of R/S research from the perspective of these thought leaders in epidemiology. Results of this study provide a clear roadmap for future R/S research investigating the impact of R/S on disease etiology in the context of U.S. prospective cohort studies.

## Conclusion

The sub-field of R/S research that focuses on the role of R/S in disease etiology faces a critical challenge. Cohort PIs state that they will only collect more R/S measures when they see a high-quality epidemiological evidence base demonstrating important influences of R/S on health, yet building this evidence base to the level of rigor required actually necessitates the availability of R/S measures within prospective cohort studies. One example that offers some hope in this regard is the recently established National Consortium on Stress, Spirituality, and Health (CoSSH), which brings together survey and clinical data from approximately 5000 cohort study adult participants across five cohort studies representing five different racial/ethnic communities: Blacks (Black Women’s Health Study), Hispanics/Latinos (Hispanic Community Health Study/Study of Latinos), American Indians (Strong Heart Study), South Asians (MASALA) and whites (Nurses’ Health Study 2). CoSSH has developed a conceptually robust and psychometrically sound survey instrument that measures a diverse set of R/S measures and was administered among these 5000 participants. These data are currently being analyzed for mechanistic analyses that investigate the impact of R/S variables on stress and health outcomes, using epigenomics, proteomics, and other biomarker data to explore plausible mechanisms of effect. Future research studies of this kind – across an even greater diversity of cohort participants, using robust biological analyses and larger sample sizes – will contribute significantly to advancing the evidence base concerning R/S influences on health.

As more prospective analyses from this and other efforts demonstrate clinically significant effects of R/S on important health conditions, including biomarkers to demonstrate a mechanism of effect, PIs may become invested in collecting a limited set of R/S measures in unison across cohort studies. Such a development could usher in a new generation of research investigating the complex ways in which individuals’ R/S beliefs and practices intersect with health and risk of disease, but only if the NIH begins funding R/S research at the level of other important psychosocial influences affecting human health. While many NIH studies have been funded that use places of worship to recruit research participants or deliver health interventions, Requests for Applications (RFAs) specifically addressing the role of R/S influences in disease etiology have been lacking. Given the high levels of religiosity among low-income and minority communities in the U.S., and the concomitant high prevalence of chronic illness among these groups, funding to support studies aimed at better understanding the role of R/S in health among these communities may be especially important in identifying new leverage points for addressing health disparities.

## Supplementary information

**Additional file 1: Table S1.** Qualitative Themes of PIs’ Perceptions of the Importance of Religion and Spirituality to Understanding Human Health. **Table S2.** Qualitative themes of PIs’ Rationales for Including or Excluding Measures of Religion and Spirituality in Cohort Studies. **Table S3.** Qualitative Themes of PIs’ Perceptions of How R/S Influences Health Outcomes. **Table S4.** Qualitative Themes of PIs’ Perceptions of What Would Motivate Them to Include R/S Measures in Future Data Collection Efforts.

## Data Availability

Transcripts of the full interviews with cohort PIs collected and qualitatively analysed in the current study are not available due to the ease with which study participants could be identified. Redacted transcripts can be made available upon request.

## Abbreviations

PI: Principal Investigator
R/S: Religion and/or spirituality
NIH: National Institutes of Health
GWAS: genome-wide association study
CoSSH: National Consortium on Psychosocial Stress, Spirituality, and Health
PHRC: Partners Human Research Committee
