# Supplementary material for: Building towards common psychosocial measures in U.S. cohort studies: principal investigators’ views regarding the role of religiosity and spirituality in human health
Source: BMC Public Health. 2020 Jun 22;20:973. doi: 10.1186/s12889-020-08854-8 (PMC7310072; doi:10.1186/s12889-020-08854-8)
Supplement: Supplementary file 1 — Additional file 1: Table S1. Qualitative Themes of PIs’ Perceptions of the Importance of Religion and Spirituality to Understanding Human Health. Table S2. Qualitative themes of PIs’ Rationales for Including or Excluding Measures of Religion and Spirituality in Cohort Studies. Table S3. Qualitative Themes of PIs’ Perceptions of How R/S Influences Health Outcomes. Table S4. Qualitative Themes of PIs’ Perceptions of What Would Motivate Them to Include R/S Measures in Future Data Collection Efforts. [file 12889_2020_8854_MOESM1_ESM.docx]

**SUPPLEMENTARY TABLES**

**Table S1.** Qualitative Themes of PIs’ Perceptions of the Importance of Religion and Spirituality to Understanding Human Health

| **Theme and Subthemes** | **Representative Quotes** |
| --- | --- |
| **Theme 1. R/S is important to human health** |  |
| R/S as a critical locus for identity and resilience | *As an epidemiologist, I think of [spirituality] primarily as a modulator of stress, but it’s more than that. There are positive social networks, and support, and healing connected with [spirituality], and sort of self-affirmation about who I am, what I’m doing, that almost for sure have physiological effects. So I look at [spirituality] as part of complex physiological networks dealing with pathways that are almost for sure related to cardiovascular disease.* |
| R/S as source of communal support | *The social support people get from church is tremendous. My own personal experience [as someone who is agnostic] makes it hard for me to judge what the added coping and support and well-being people get from their religious beliefs is, as opposed to the social framework. But for me, you know personally, going to a synagogue when [experiencing a personally difficult time] was a tremendous social boon.…And so I do believe that the support that comes from [being part of a religious community] allows people to cope better with challenges, to be less overwrought, to help them get things done, to reinforce messages about healthy lifestyle. So you know, from that perspective, I think it’s a nobrainer [that R/S affects health].* |
| R/S promotes healthy behaviors | *Seventh Day Adventists have a whole pattern of behavior based on their religion that influences their risk of disease, as do people of Eastern sects, in Hindus and other groups, have very specific sorts of prescribed behavior patterns based on religion. So it can be very powerful.* |
| R/S has differential effects on distinct racial/ethnic communities | *I think it would be more important to study [religion and spirituality] in minority groups, who are under other stressors. [Religion and spirituality] would be an important factor for… minority groups in general. We’re putting more emphasis on [religion and spirituality] as factors that moderate stressors or factors that could turn things around, and thus improving the quality of life or life expenctancy, or even prevent the recurrence of certain diseases…So for African Americans, religious activities are really, really important.* |
| R/S research is hampered by barriers (e.g., bias and limitations of measures) | *So though the question of spirituality might seem reasonable to us, spirituality is something that many members of my [research] team might go, ‘Blah!’* |
| **Theme 2. R/S may or may not be important to human health** |  |
| Insufficient evidence to determine if R/S influences health | *I think the findings on spirituality measures have been sort of mixed in relation to disease outcomes. Well, that’s the nature of our field, isn’t it? A lot of factors, when you look at the data, are mixed! And it’s only after a crucial body of evidence has emerged that you’re able to look at the totality of studies. So right now, the field is not mature. You don’t have a lot of studies in this area, and findings are mixed.* |
| Lack of a conceptual framework for how R/S influences health outcomes | As *you know, CVD is a lifecourse disease, and it’s a lifestyle disease. You know, biologically, we understand that here’s coronary plaque, it’s a vulnerable plaque, an ulcerated plaque, and on top of that, there’s a superimposed thrombosis. So we understand the biological mechanisms. But for each of those steps, why is the plaque vulnerable? Why is there a plaque in the first place? – you know, the underlying vessel, the wall, and endothelial function, the hemostatic factors, the fibrinolytic factors? And we understand all this is a constellation of biological phenomena that translate into an event on a particular day, in a particular time, in a particular moment in a person’s life. But, you know, it follows if you look back – there’s a cascade of events, which begins with the genetics that you are born with, and a series of environmental factors that you’re exposed to, including the built environment, and including your socioeconomic position over the life course. And on top of that, a number of bio-behavioral risk factors. So religiosity is one of a gamut of [influences] on a complex risk factor. So I think one of the challenges is going to be to build a framework, a thesis, or a scientific model that places religiosity in the context of a lot of other pathways that eventually condenses into a biological model.* |
| **Theme 3. R/S is not important to human health** |  |
| Lack of compelling research linking R/S and health, largely due to conflicting findings | *My recollection of the [R/S] literature is that the findings are all over the place.* |
| R/S measures are proxies of other, more important factors influencing health | *I don’t feel like, personally, that these [R/S] variables are very important variables to be looking at – if anything, they are proxies of something else that I would prefer to get at. So if they’re proxies of social support, I’d prefer to get a measure of social support. If they’re proxies for stress mitigation, I’d like to get at more of a proxy of stress. So I feel [R/S] is like a third variable that out there as a proxy, and not really our exposure of interest.* |
| No plausible mechanisms through which R/S might influence health | *This exposure couldn’t possibly cause or protect one from cancer, therefore we don’t see this [the role of R/S in health] as a reasonable scientific question.* |
| R/S research can’t lead to targetable interventions | *I mean, what are you going to do? Prescribe that people start going to church?* |

**Table S2.** Qualitative themes of PIs’ Rationales for Including or Excluding Measures of Religion and Spirituality in Cohort Studies

| **Theme and Subthemes** | **Representative Quotes** |
| --- | --- |
| **Theme 1. Rationales for including R/S measures in cohort studies** |  |
| Data supporting relationship between R/S and health as rationale for inclusion of R/S measures | *You know, the other concept that we had, which I think is still valid, is that religion could be considered in one sense to be the parent, or if you like, to be behind behaviors. In other words, religion is driving the dietary behaviors in the path, which in turn results in some differences in chronic disease.* |
| Inclusion of R/S measures being the result of advocacy by are researcher with an interest in the topic | *In every case, you have advocates. You have folks who say, here’s our rationale. Here’s why we think it’s important . And I think that probably [name] was the advocate for [our R/S measures}. And, you know, just thinking about quality of life, thinking about social support, thinking about the importance of spiritualty in a global sense, of how it would contribute to us really understanding not only the sort of overarching factors associated with things like cardiovascular disease, but also recognizing that we have a very diverse cohort in which spirituality may mediate or may be protective differentially in one of our racial/ethnic groups. So that’s the overall rationale for inclusion of spirituality [measures].* |
| Rationale for inclusion of R/S measures being the salient role that R/S has for the racial/ethnic minority groups being studied | *I think again, you can’t speak for all African Americans, but in general, particularly in the South, you are speaking about a society that sees religion and spirituality as a central aspect of life. And that, I think, has been true for generations, and is impressively true today. I think if you were to ask 100 African Americans in the South whether they attend church regularly, I would say that 85 of them would say yes… And I think most feel that any complete healthy life includes a good amount of attention to spirituality, that one of the great methods of dealing with stress is to turn to turn to God, to turn to a higher power…These are very, very important ideas and ways of life, really. And [people in the South] do seem them as ways of coping, but they see, beyond that, that this is fundamental to how life should be lived.* |
| **Theme 2. Rationales for excluding R/S measures from cohort studies** |  |
| R/S evidence is not sufficiently rigorous to warrant inclustion | *You’d expect social support to have an impact on a number of domains. But, from my vantage point, I’ve not seen evidence that [R/S] is at least very strong at this point in time.* |
| Lack of inclusion of R/S measures by the research team due to personal biases | *[R/S measures aren’t included] for one thing, because most of the investigators are biologically inclined – you know, to look for more biological or genetic explanations of things…. My anecdotal impression – and this based on myself, really – is that they [PIs] are not religious. Probably there’s a bigger percentage of naysayers [about the importance of R/S to health] among scientist investigators than maybe other groups of people.* |
| The study of the role of R/S in health can’t lead to targetable interventions | *So this is where personal bias comes in….We look at poverty and mortality, but we can’t stop there, because we are saying, “how does poverty affect mortality?” I can’t make everybody rich, but I can look at the pathways that poverty works through to influence mortality – maybe diet, maybe stress, maybe lack of access to healthcare, and things like that. We can fix those things. [Regarding church attendance and mortality], I just wonder what the message is….Is the message that people should find God? Or go to church more often? From a personal background, I would feel uncomfortable with public health messages that had to do with religion.* |
| Lack of funding to support inclusion of R/S measures into public health research | *We had no funding for them (R/S measures); nobody was writing a specific grant related to those hypotheses.* |

**Table S3.** Qualitative Themes of PIs’ Perceptions of How R/S Influences Health Outcomes

| **Theme and Subthemes** | **Representative Quotes** |
| --- | --- |
| **Mechanisms for how R/S influences human health** |  |
| R/S as a vehicle for garnering social support | *The social support people get from church is tremendous. My own personal experience [as someone who is agnostic] makes it hard for me to judge what the added coping and support and well-being people get from their religious beliefs is, as opposed to the social framework. But for me, you know personally, going to a synagogue when I [was experiencing a personally difficult time] was a tremendous social boon… And so I do believe that the support that comes from [being part of a religious community] allows people to cope better with challenges, to be less overwrought, to help them get things done, to reinforce messages about healthy lifestyle. So you know, from that perspective, I think it’s a no-brainer [that R/S affects health].* |
| R/S as a means of coping with stress | *Probably the most immediate and obvious is related to the stress pathways. And they are related to physiology all over the body – immune function, endocrine function – that would potentially interface with pathways related to cancer, but not as directly as to cardio-metabolic outcomes, one would think. So cortisol, epinephrine, those pathways would be presumably the most immediately directly affected, whether there [via] hypothesized immune markers, which do seem to be related, secondarily at least, but perhaps primarlly to thes stress pathway. With us, one of the most importessive assocaitions we’ve seen has tbeen with the Crown-Crisp Index, which is a measure of phobic anxiatey and that is related to cardiac arrhythmia, presumably mostly biochemically similar to stress and stress pathways, but affecting arrhythmias.* |
| R/S promotes healthy behaviors | *The jury is still out [regarding how R/S affects health], but I think that one can pretty reasonably postulate that some of these [R/S beliefs or traditions] might be driving dietary behavior and so may not be confounders in that sense, but rather the dietary behavior might mediate, in part, the effect of religions variables.* |
| R/S facilitates better psychological well-being | *We were finding that a major pathway [of spirituality’s influence on health] seems to be because their religion lowers negative emotionality, and basically, we didn’t find any direct connection initially from religiosity to perceived health, but we did find an indirect effect through a reduction of negative emotionality.* |
| Mechanisms of how R/S influences health are not fully known | *Well, [stress] is certainly one pathway, but I don’t know enough about the biology to express an opinion about what other pathways may be involved. But there probably are other pathways. I mean, there always do seem to be multiple pathways* |

**Table S4.** Qualitative Themes of PIs’ Perceptions of What Would Motivate Them to Include R/S Measures in Cohort Studies

| **Themes** | **Representative Quotes** |
| --- | --- |
| High quality, prospective studies regarding R/S and health are needed | *Any more [cross-sectional] association studies will just add to the confusion.* |
| R/S research that eludicates biological mechanisms through which R/S influences health is needed | *What would be very persuasive to me would be some well-designed research that showed biological changes, or some physiologic effects, that were associated with people’s religion or spirituality, on pathways that were meaningful for cancer, cancer pathways.* |
| Valid R/S measures are needed that can be harmonized across studies | *For example, coffee is a habitual activity that a large number of people partake in. Very, very easy to measure, and thus we’ve got dozens or hundreds of studies on coffee! – almost every epidemiologist looking for something that’s either protective or is caused by coffee, though there’s no findings there...It’s easy to ask somebody what their coffee habit is. But it’s very hard to get at these kinds of things [R/S influences]…This [R/S research] is so ripe for epidemiological discovery if you can figure out how to measure it.* |
| Need to address bias within the public health research community | *So though the question of spirituality might seem reasonable to us, spirituality is something that many members of my team might go, “Blah!”* |
| Research funding needed to support examining R/S and health outcomes | *I’m not sure if people would fund it. And when I say ‘fund it’, I mean at NIH. I’ve never applied for funding anywhere except through NIH. So I think it’s hard to come by… I think many of us would be interested in doing it if we could, but the resources are hard to come by.”* |
